# SMAD2 inhibits pyroptosis of fibroblast-like synoviocytes and secretion of inflammatory factors via the TGF-β pathway in rheumatoid arthritis

**DOI:** 10.1186/s13075-023-03136-1

**Published:** 2023-08-09

**Authors:** Xingxing Mao, Weijie Wu, Yunyi Nan, Weiwei Sun, Youhua Wang

**Affiliations:** 1 Suzhou Medical College of Soochow University, Suzhou, 215000 China; 2 Department of Orthopaedics, Affiliated Hospital of Nantong University, Medical School of Nantong University, Nantong, 226001 China; 3 Department of Orthopaedics, Affiliated Nantong Hospital of Shanghai University, The Six People’s Hospital of Nantong, Nantong, Jiangsu, 226001 China

**Keywords:** SMAD2, Pyroptosis, Rheumatoid arthritis, TGF-β, Fibroblast-like synoviocytes

## Abstract

**Objective:**

Rheumatoid arthritis (RA) is a chronic, progressive autoimmune disease. Over-activation of fibroblast-like synoviocytes is responsible for the hyperplasia of synovium and destruction of cartilage and bone and pyroptosis of FLS plays a key role in those pathological processes during RA. This study investigated the detailed mechanisms that SMAD2 regulates the pyroptosis of FLS and secretion of inflammatory factors in rheumatoid arthritis.

**Methods:**

We collected synovial tissues of RA patients and FLS-RA and cultured FLS for detection of expression of SMAD2. ASC, NLRP3, cleaved-caspase-1, and GSDMD-N were detected by Western blot after overexpression of SMAD2. Besides, flow cytometry, electron microscope, ELISA, HE staining, and Safranin O staining were performed to further demonstrate that SMAD2 can affect the pyroptosis of FLS-RA.

**Results:**

The expression of SMAD2 was down-regulated in synovial tissues of RA patients and FLS-RA. Overexpression of SMAD2 can inhibit the expression of ASC, NLRP3, cleaved-caspase-1, and GSDMD-N. Flow cytometry and electron microscope further demonstrated that SMAD2 attenuated pyroptosis of FLS-RA. In addition, overexpression of SMAD2 also inhibited inflammatory factors such as IL-1β, IL-18, IL-6, and IL-8 secretion and release of LDH. Besides, overexpression of SMAD2 can reverse the decrease of p-SMAD2 and TGF-TGF-β induced by nigericin. In vivo experiments on CIA rats further demonstrated that overexpression of SMAD2 by local intra-articular injection of LV-SMAD2 can effectively alleviate joint redness, swelling, and destruction of cartilage and bones.

**Conclusion:**

SMAD2 inhibited FLS-RA pyroptosis by down-regulating of NLRP3 inflammasomes (NLRP3, ASC, and caspase-1 complex) and eased the secretion of inflammatory factors via the TGF-β signaling pathway, thereby improving the symptom of RA. We hope that this study may provide a new research idea for RA and a potential target for the treatment of RA.

**Supplementary Information:**

The online version contains supplementary material available at 10.1186/s13075-023-03136-1.

## Introduction

Rheumatoid arthritis (RA) is a chronic autoimmune disease characterized by systemic inflammation response and persistent synovitis, which lead to swelling of peripheral joints, hyperplasia of joint synovium, and destruction of cartilage and bone [1]. Approximately 0.5–1% of adults worldwide suffer from RA, resulting in heavy economic burdens to society and patients [2]. It is reported that abnormal activation of fibroblast-like synoviocytes (FLS) is an important event of RA [3]. FLS is the main cell in the lining layer of synovial, which has a lot of tumor-like characteristics, such as enhanced abilities of proliferation, migration, and invasion [4, 5]. Besides, FLS-RA secrete pro-inflammation cytokines including interleukin (IL)-6, IL-1β, and so on, contributing to joint swelling and destruction of cartilage and bone [6]. Therefore, inhibition of over-activated FLS-RA is the traditional strategy for RA treatment, including disease-modifying anti-rheumatic drugs (DMARDs), while these drugs have limited benefits and serious side effects in clinical [7–9]. Above all indicating that there are other unknown mechanisms during the pathogenesis of RA.

Pyroptosis is a form of programmed cell death triggered by nucleotide-binding oligomerization segment-like receptor family 3 (NLRP3) inflammasomes [10]. NLRP3 inflammasomes recruit and activate caspase-1 through adaptor molecule ASC. Activated caspase-1 cleave gasdermin-D (GSDMD) into N-terminal pore-forming domain (PFD) and C-terminal repressor domain (RD). PFD result in the formation of pores on the cell membrane, leading to cell swelling, cell membrane rupture, and lactate dehydrogenase (LDH) secretion [11]. On the other hand, activated caspase-1 can also transform IL-1β and IL-18 to mature forms and trigger pyroptotic cell death subsequently [12]. It is reported that IL-1β and IL-18 are elevated in synovitis tissue of RA patients [13], which indicates that pyroptosis is involved in the occurrence and development of RA. FLS is an important cell type in synovial tissue, the level of pyroptosis was elevated in FLS isolated from RA patients compared with non-RA patients, and this process is closely related to NLRP3 inflammasomes (NLRP3, ASC, and caspase-1 complex) [14]. Research on pyroptosis of FLS-RA may provide effective therapeutic strategies in the future.

The transforming growth factor β (TGF-β) signal pathway is pivotal in the transcription of multiple gene and cell function [15, 16], which has been demonstrated that the TGF-β signal pathway is closely related to osteoarthritis (OA). SMAD family member 2 (SMAD2) as a downstream molecule of TGF-β, plays a key role in transporting extracellular signals from TGF-β ligands into the nucleus, which causes a large amount of biological processes, such as cell proliferation and apoptosis [17]. Gen Li et al. reported that miR-155 inhibits chondrocyte pyroptosis by targeting SMAD2 and inhibiting the NLRP3/Caspase-1 to protect against OA [18]. Li Z et al. also reported that activation of SMAD2/3 can effectively inhibit the expression of NLRP3, reducing the pyroptosis of hypoxic cardiomyocytes [19], whether SMAD2 participates in the pyroptosis of FLS-RA during RA has not been cleared. In this study, we firstly demonstrated that SMAD2 was down-expressed in FLS-RA, and overexpression of SMAD2 can inhibit NLRP3/Caspase-1 pathway, thus attenuating the symptoms in collagen-induced arthritis (CIA) rats.

## Materials and methods

### Establishment of the CIA rat model

Six-week-old male Wistar rats (approximately 180–200 g) were purchased from the Laboratory Animal Center of Nantong University. The Animal Ethics Committee has approved all animal experiments (including rat anesthesia and euthanasia procedures) of Nantong University. Animals were housed in a 12-h light-dark cycle under relative humidity and temperature conditions of 22 ± 3 °C SPF, fed with a regular diet and water for two weeks, and then the CIA model was induced. Type II bovine collagen was dissolved in 0.1 N acetic acid solution at a concentration of 10 mg/mL, and then complete Freund’s adjuvant was emulsified with collagen solution. 200 μl collagen-adjuvant emulsion (1:1) was injected into the base of the rat’s tail by intradermal injection. One hundred microliters of immunopotentiator was injected at the same site in the same way 1 week later. The control rats were subcutaneously injected with the same amount of saline at the same time. Arthritis index (AI) was used to evaluate the severity of arthritis in each rat: 0 for no swelling, 1 for interphalangeal joint involvement, 2 for mild joint and toe swelling, 3 for toe and ankle swelling, and 4 for severe arthritis of the entire foot point. Scoring was performed based on the condition of four paws to obtain a total clinical score of 16 points per rat. Generally, the value of AI ≥ 4 represented the successful establishment of the RA rat model. Four weeks after the first immunization, rats were randomly divided into the normal group (rats injected with saline, *n* = 5), CIA group (collagen-induced rats, *n* = 5), NC group (intra-articular injection of negative control lentivirus RA rats, *n* = 5), CIA+SMAD2 group ((intra-articular injection of Plv3-CMV-Smad2 (rat) -3×FLAG-CopGFP-Puro lentivirus RA rats, *n* = 5), Each group of rats was scored for arthritis, and paw thickness was determined using vernier calipers once a week. On the 49th day, the rats were sacrificed, serum was used for the detection of inflammatory factors by ELISA kit and ankle joints were taken out for H&E staining and Safranin O staining respectively.

### Information of lentiviral vectors

We purchased lentiviral vectors from miaolingbio, a Chinese biotechnology company providing plasmid sharing and lentivirus packaging services. During the preparation of lentivirus, 293 T cells were used as packaging cells and the lipofection reagents was PEI compounded by miaolingbio themselves.

### Isolation of FLSs

Fresh synovial tissues obtained from patients of knee arthroplasty of RA or severe joint trauma were washed three times with D-PBS(Beyotime) supplemented with 10% penicillin-streptomycin solution(Beyotime) (1200 r/min, centrifugation for 6 min), cut into 1 mm pieces with sterile scissors, and transferred to 1 mg/ml collagenase II (Gbico), gently pipetting to resuspend the tissue block, put it into a 37-degree constant temperature shaking incubator for 4 h, and then add DMEM-F12 medium (Gbico) supplemented with 20% fetal bovine serum and 1% penicillin-streptomycin solution. Centrifuge (1500 r/min, 10 min), discard the supernatant, resuspend the cells with DMEM-F12 medium, and transfer them into a culture flask. FLS were confirmed by positive staining for Vimentin with a fibroblast-like synovial cell marker. Cells in passages 3–6 were used in this study.

### ELISA assays

The supernatants originated from FLS thawed at room temperature, and concentrations of IL-1β (EK101B-01, multi sciences, China), IL-18 (EK118-02, multi sciences, China), IL-6(EK106/2-01, multi sciences, China), and IL-8 (EK108-03, multi sciences, China) were determined by ELISA kits according to the manufacturer’s protocol.

### LDH release assay

LDH release assay was measured using the CyQUANT LDH Kit (C20300, Invitrogen) according to the manufacturer’s instructions. The absorbance value was measured at 490 nm and 630 nm.

### RT-PCR

According to the manufacturer's instructions, total RNA was prepared from synovial tissue or FLS using the RNA-Quick Purification Kit (Shanghai Yishan Bio). The obtained RNA was reverse transcribed into cDNA using SweScript cDNA Synthesis SuperMix (Servicebio). cDNA was diluted 1:5 before being used for real-time quantitative polymerase chain reaction (Q-PCR) analysis. The sequence of SMAD2 was (Forward ctcttctggctcagtctgttaa, Reverse aaggagtacttgttaccgtctg). Target genes were subjected to qRT-PCR analysis in 20 μL reactions containing SYBR Green (10 μL, Servicebio), primers (1 μL each of forwarding and reverse primers), cDNA template (2.0 μL), and ddH2O (6.0 μL). Target mRNA expression in each sample was normalized to GAPDH expression compared to control samples.

### Safranin O-fast green staining and hematoxylin-eosin (HE) staining

Safranin O-fast green staining was performed according to the manufacturer’s instructions (G1371 Solarbio). For HE staining, sections were deparaffinized, hydrated, and stained with hematoxylin for 10 min. After PBS washing, the sections were counterstained with eosin, dehydrated with ethanol, cleared with xylene, mounted with neutral gum, and observed under a microscope. At least three sections from the same site were observed in each group.

### Western blotting analysis

Proteins were subjected to 10% sodium dodecyl sulfate–polyacrylamide gel electrophoresis (SDS-PAGE) and then transferred onto a PVDF membrane. The membranes were blocked with Ncm Blot Blocking Buffer for 15min at room temperature, followed by incubation with anti-SMAD2 (67343-1-Ig, proteintech), anti-Phospho-SMAD2 (#3108, Cell Signal Technology), anti-GSDMD (20770-1-AP proteintech), anti-ASC (67494-1-Ig, proteintech), anti-NLRP3 (#13158, Cell Signaling Technology) and GAPDH antibody (60004-1-Ig, proteintech) overnight at 4 °C. The membranes were washed three times and incubated with secondary anti-rabbit IgG (SA00001-2, proteintech) or anti-mouse IgG (SA00001-1, proteintech) for 1 h. Protein bands were visualized on a Western blotting detection system (Bio-Rad, USA).

### Flow cytometry

The APC Annexin V kit (#640930, Biolegend) was used to detect cell death according to the manufacturer's instructions. Cells were harvested and incubated with Annexin V and 7-AAD antibody in 1 × Annexin V binding buffer for 15 min at room temperature. After staining, 300 μl 1 × binding buffer was added, and the cells were immediately analyzed. Pyroptosis caspase-1 assay was performed according to the manufacturer’s instructions (#9158, Immunochemistry Technologies).

### Statistical analysis

All graphs and statistical data were generated using GraphPad Prism 7.0 software. One-way analysis of variance (ANOVA) was used to compare differences among groups for data with a normal distribution and homogeneity of variance.

## Result

### SMAD2 level was decreased in RA patients

**Fig. 1:**
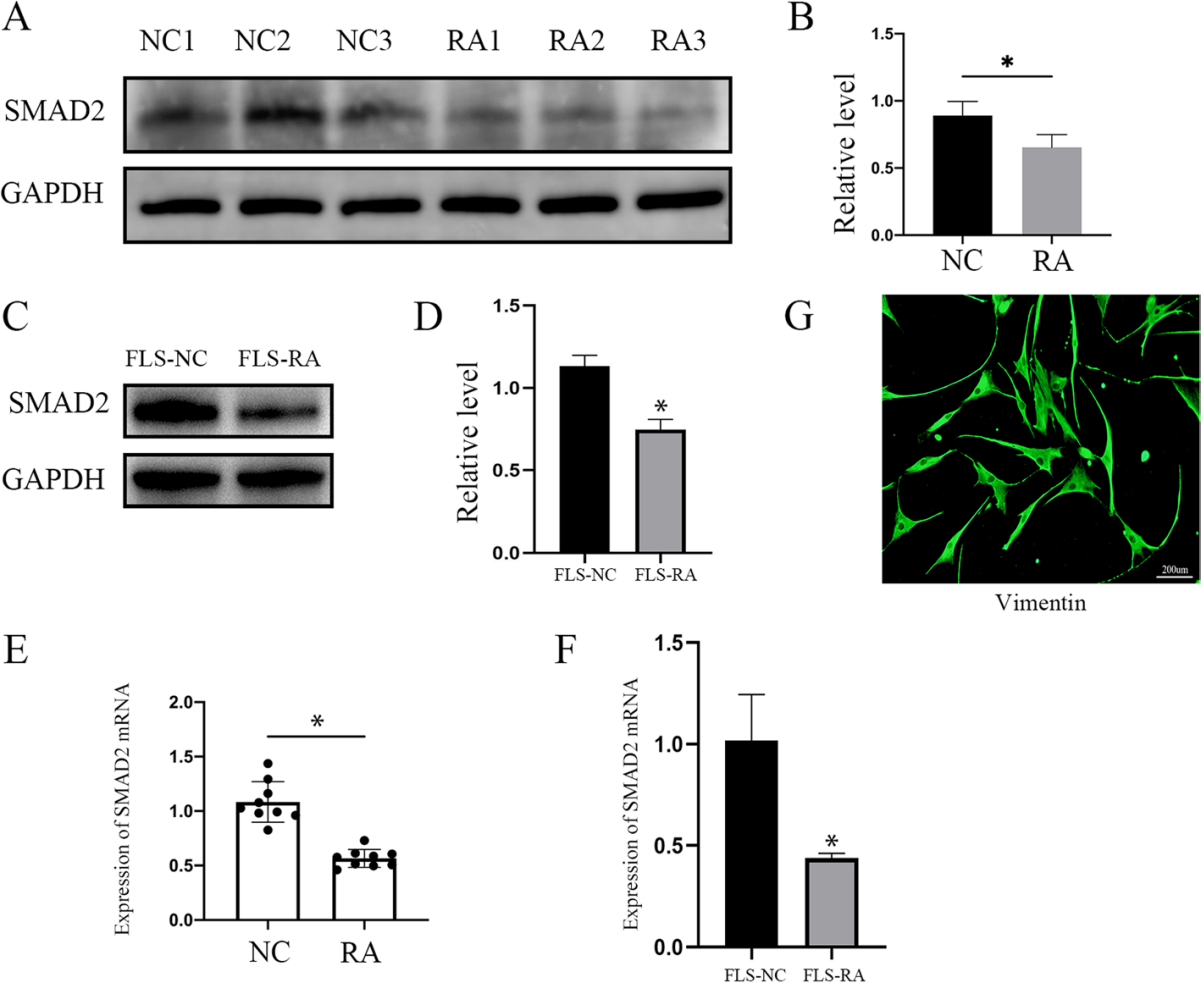
SMAD2 level was decreased in RA patients. **A**, **B**, **E** SMAD2 expression in synovial tissues from RA patients and non-RA patients was detected by Western blot and RT-PCR. **C**, **D**, **F** SMAD2 expression in FLS isolated from RA patients (FLS-RA) and non-RA patients (FLS-NC) was detected by Western blot and RT-PCR. **G** Positive marker (Vimentin) of FLS was detected by immunofluorescence. The experiment was repeated three times and statistical data was presented as mean ± SD. * *P*<0.05

To investigate the expression level of SMAD2 in RA patients, we collected synovial tissues from RA patients during total knee arthroplasty surgery and non-RA patients undergoing severe joint trauma. The expression of SMAD2 was decreased in RA patients’ synovial tissues compared with control groups (Fig. 1A, B; *P*<0.05). SMAD2 mRNA level was significantly decreased in RA groups compared with control groups (Fig. 1E; *P*<0.05). FLS as the main cell type in the lining layer of synovial, play a key role in the development of RA. We isolated and cultured primary FLS, Western blot, and RT-PCR results also showed that SMAD2 was downregulated in FLS-RA (Fig. 1C, D, F; *P*<0.05). Besides, FLS were confirmed by positive staining for Vimentin with a fibroblast-like synovial cell marker (Fig. 1G).

### SMAD2 affected the expression of ASC, NLRP3, Caspase-1, and GSDMD

**Fig. 2:**
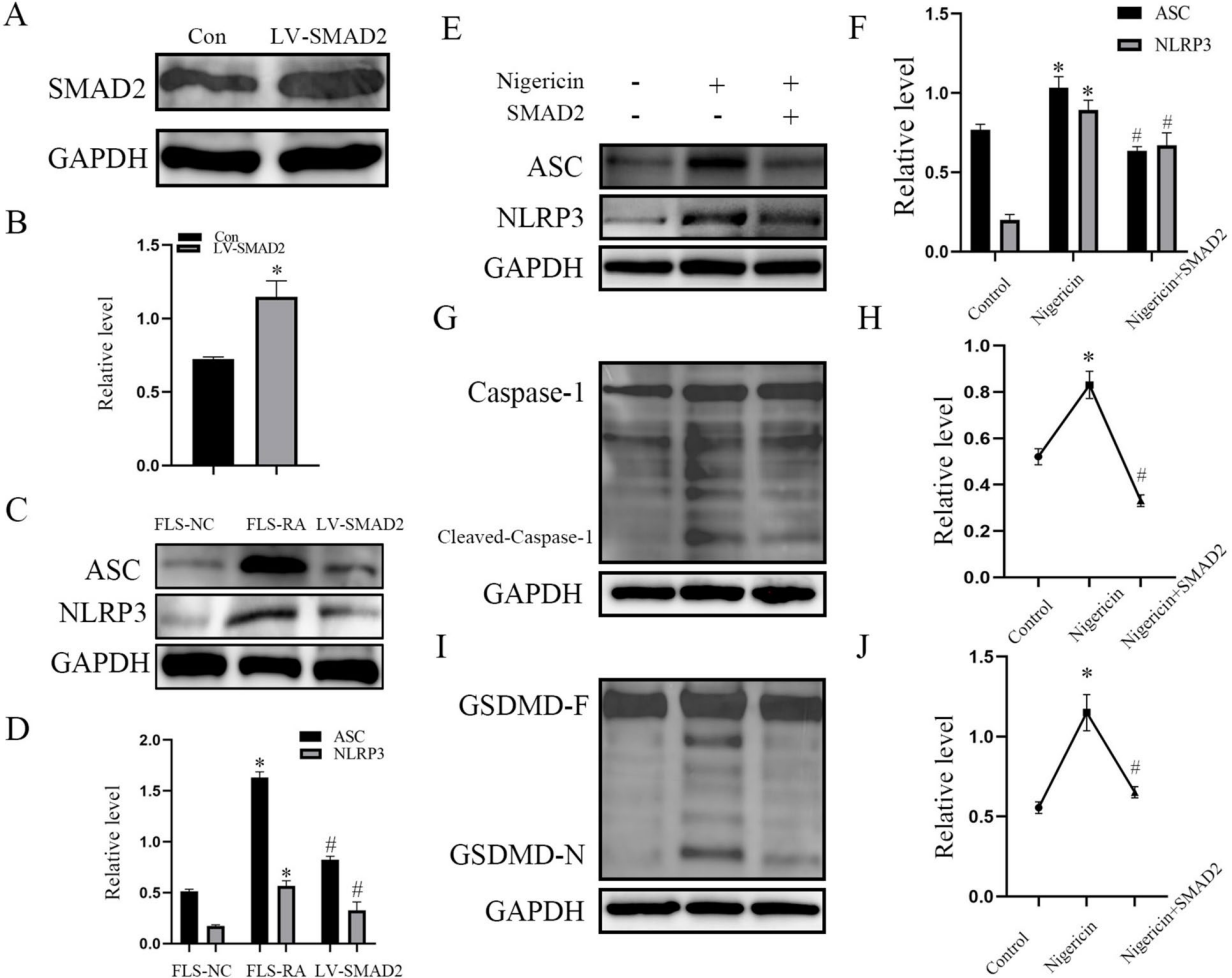
SMAD2 affected the expression of ASC, NLRP3, Caspase-1, and GSDMD. **A**, **B** LV-SMAD2 was transfected into FLS-RA, and the expression of SMAD2 was detected by Western blot. **C**, **D** LV-SMAD2 was transfected into FLS-RA, and the expression of ASC and NLRP3 was detected in FLS-NC, FLS-RA, and LV-SMAD2.** E**, **F** FLS-RA were treated with nigericin by concentration of 10 uM for 24h accompanied with LV-SMAD2 or not, the expression of ASC, NLRP3 were detected by Western blot.** G**–**J** the expression of caspase-1, cleaved-caspase-1, GSDMD-Full, and GSDMD-N in three groups were detected by Western blot. The experiment was repeated three times and statistical data was presented as mean ± SD. In **D**, * means comparison between FLS-NC and FLS-RA groups. # means comparison between FLS-NC and LV-SMAD2 groups. * ,# *P*<0.05. In **F**, **H**, and **J**, * means comparison between control and nigericin groups. # means comparison between nigericin and nigericin+SMAD2 groups. * ,# *P*<0.05

FLS pyroptosis plays a crucial role in the entire pathogenesis of RA, we hypothesized that downregulated SMAD2 in FLS-RA may participate in FLS pyroptosis. Firstly, we conducted LV-SMAD2 and transfected into FLS-RA, the expression of SMAD2 was up-regulated (Fig. 2A, B). Besides, the expression of ASC and NLRP3 were higher in FLS derived from RA patients compared to FLS derived from healthy individuals and rescued by overexpression of SMAD2 (Fig. 2C, D), then we induced FLS-RA pyroptosis with nigericin (10 nM) for 24h. Compared with nigericin treated group, ASC and NLRP3 displayed decreased expression in the LV-SMAD2-treated group (Fig. 2E, F; *P*<0.05). In three groups, the level of caspase-1 and GSDMD-Full did not change significantly, while after being treated with nigericin and LV-SMAD2, cleaved-caspase-1 and GSDMD-N terminal decreased compared with the nigericin-treated group (Fig. 2G–J; *P*<0.05). Above results indicated that overexpression of SMAD2 can attenuate pyroptosis of FLS-RA induced by nigericin.

### SMAD2 affected FLS-RA pyroptosis

**Fig. 3:**
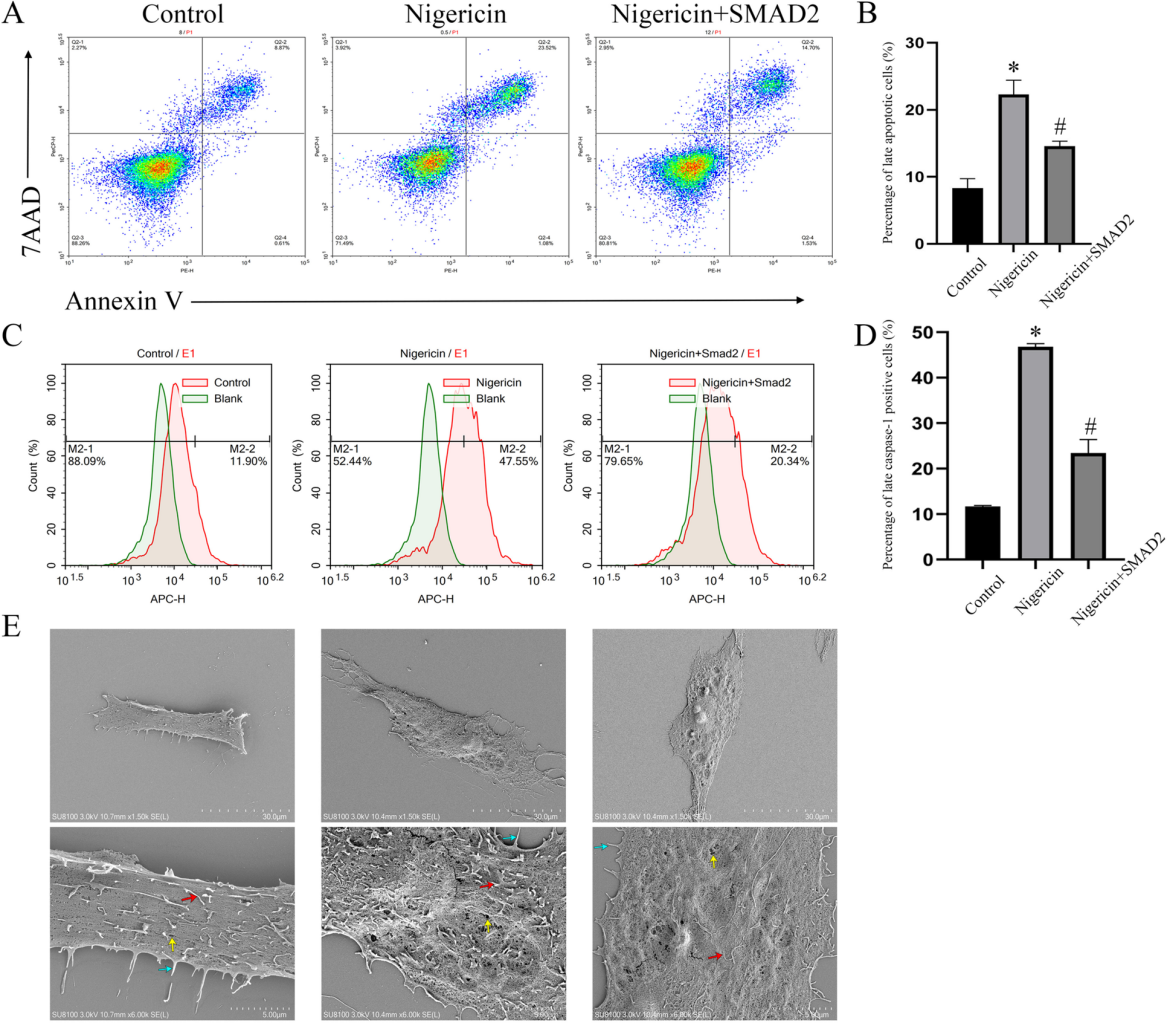
Overexpression of SMAD2 alleviated FLS-RA pyroptosis. **A**, **B** Apoptosis rate of FLS-RA was detected by flow cytometry in three groups. **C**, **D** The percentage of caspase-1 positive FLS was detected by flow cytometry in three groups. **E** Electron microscope of three groups of FLS. The green arrow means pseudopodia. The yellow arrow means small pores. The red arrow means microvilli. The experiment was repeated three times and statistical data was presented as mean ± SD. In **B**, **D**, * means comparison between control and nigericin groups. # means comparison between nigericin and nigericin+SMAD2 groups. * ,# P<0.05

**Fig. 4:**
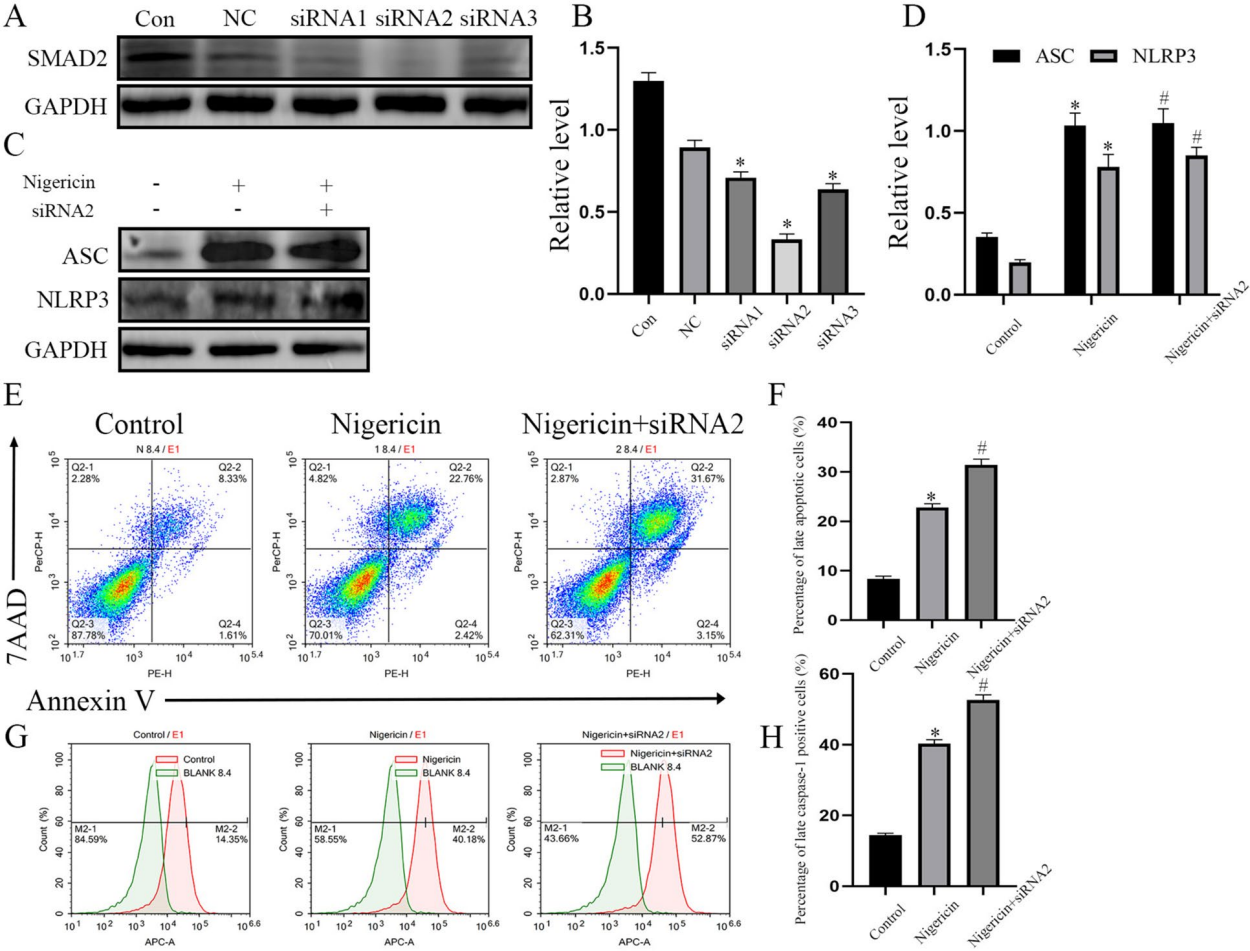
Downregulation of SMAD2 aggravates FLS-RA pyroptosis. **A**, **B** siRNA-SMAD2 was transfected into FLS-RA, and the expression of SMAD2 was detected by Western blot (NC stands for negative control siRNA). **C**, **D** FLS-RA were treated with nigericin by the concentration of 10 µM for 24h accompanied with siRNA-SMAD2 or not, the expression of ASC, NLRP3 were detected by Western blot.** E**, **F** Apoptosis rate of FLS-RA was detected by flow cytometry in three groups. **G**, **H** The percentage of caspase-1 positive FLS was detected by flow cytometry in three groups. The experiment was repeated three times and statistical data was presented as mean ± SD. In **B**, * means comparison between control and siRNA2 groups. In Fig4D.F.H, * means comparison between Control and Nigericin groups. # means comparison between nigericin and nigericin+siRNA2 groups. * ,# *P*<0.05

Next, we performed a flow cytometry analysis. The results showed that the percentage of 7− AAD+ annexin V+ late apoptotic cells among FLSs from the nigericin- and LV-SMAD2-treated group was significantly decreased compared with nigericin treatment alone (Fig. 3A, B; *P*<0.05). The pyroptosis of FLS-RA was marked with positive for FITC anticaspase-1, and the ratio of pyroptosis was blocked in the nigericin- and LV-SMAD2-treated group compared with the nigericin-treated group (Fig. 3C, D; *P*<0.05). To further prove the influence of SMAD2 on FLS-RA pyroptosis, we scanned FLSs with an electron microscope, there are fewer small pores, thinner microvilli on the cell membrane, and shorter pseudopodia in the nigericin- and LV-SMAD2-treated group than that in nigericin treated group (Fig. 3E; *P*<0.05). In addition, we also transfected siRNA of SMAD2 into FLS-RA to further demonstrated the above results, the detail sequence information about siRNA were shown in Table 1. As shown in Fig. 4, the down-regulation of SMAD2 did not further increase the expression of ASC and NLRP3 compared with that in the nigericin-treated group significantly (Fig. 4A–D). We analyzed the results and speculated that SMAD2 expression is already downregulated by nigericin treatment and that it would be difficult to detect additional effects of siRNA treatment. Therefore, the difference in ASC and NLRP3 expression between the nigericin-treated and nigericin+siRNA-treated groups might not appear significant. Besides, flow cytometry analysis also presented that the percentage of 7− AAD+ annexin V+ late apoptotic cells and caspase-1 positive cells was higher in nigericin and siRNA-SMAD2-treated group than in the nigericin-only-treated group (Fig. 4E, H).

**Table 1:** The detail sequence information about siRNA in Figure4. Negative control means NC, SiRNA1 homo-1067 means siRNA1, SiRNA2 homo-1353 means siRNA2, SiRNA3 homo-480 means siRNA3

**Negative control**	**sense 5′-UUC UCC GAA CGU GUC ACG UTT-3′**
	**antisense 5′-ACG UGA CAC GUU CGG AGA ATT-3′**
**SiRNA1 homo-1067**	**sense 5′-GGUGUUCGAUAGCAUAUUATT-3′**
	**antisense 5′-UAAUAUGCUAUCGAACACCTT-3′**
**SiRNA2 homo-1353**	**sense 5′-CCCUGCAACAGUGUGUAAATT-3′**
	**antisense 5′-UUUACACACUGUUGCAGGGTT-3′**
**SiRNA3 homo-480**	**sense 5′-CCAAGCACUUGCUCUGAAATT-3′**
	**antisense 5′-UUUCAGAGCAAGUGCUUGGTT-3′**

### Overexpression of SMAD2 alleviated secretion of inflammatory factors and release of LDH

**Fig. 5:**
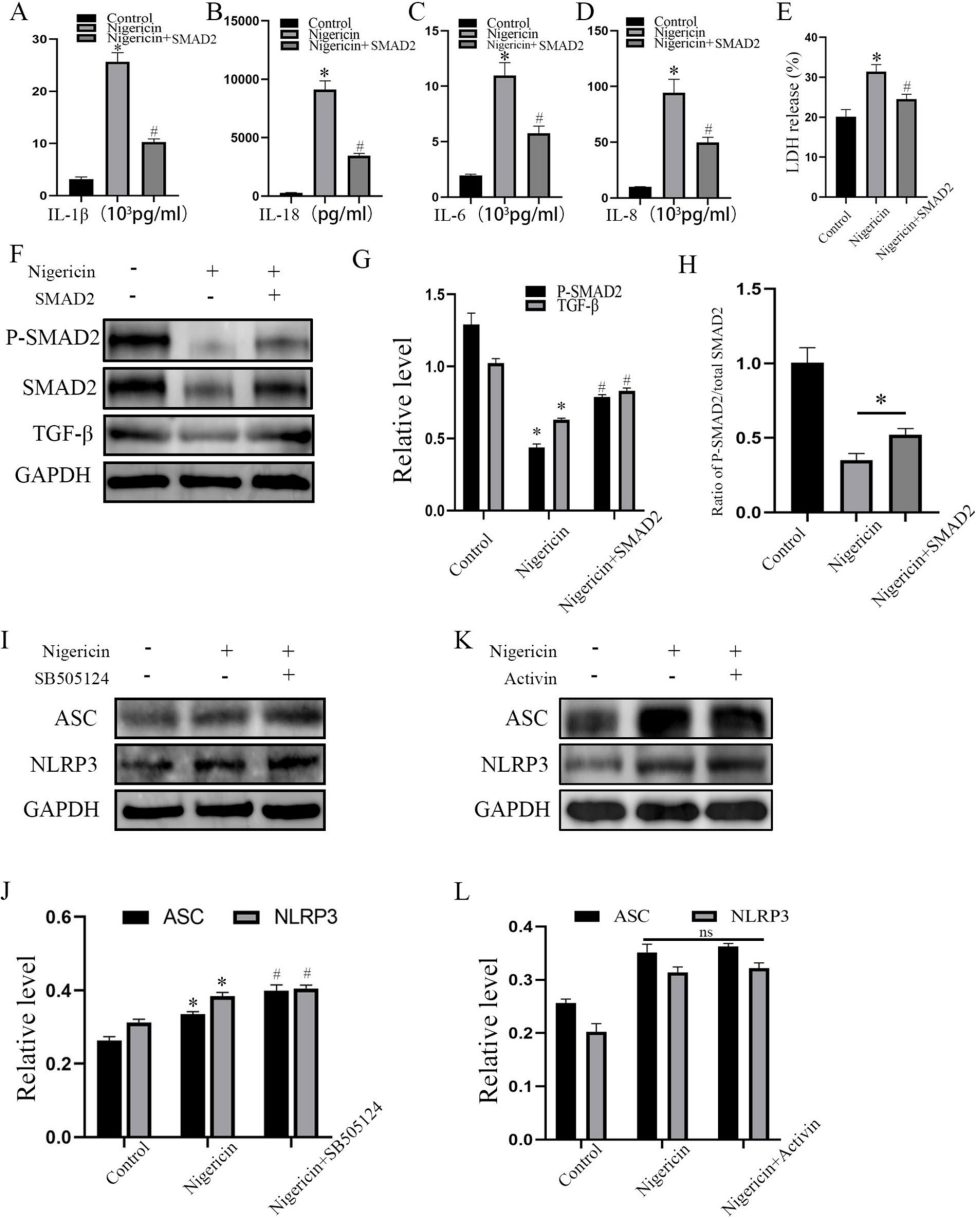
SMAD2 inhibited secretion of inflammatory factors and affected FLS-RA pyroptosis via the TGF-β pathway. **A–D** Content of inflammatory cytokines IL-1β, IL-18, IL-6, and IL-8 in FLS-RA supernatant were determined by ELISA. **E** The concentration of LDH was detected. **F**, **G** FLS-RA were treated with nigericin by concentration of 10 uM for 24h accompanied with LV-SMAD2 or not, the expression of P-SMAD2, total SMAD2, TGF-β were detected by Western blot. **H** Ratio of p-SMAD2/total SMAD2. **I**, **J** FLS-RA were treated with nigericin by the concentration of 10 µM for 24h accompanied with SB505124 (inhibitor of TGF-β receptor I) or not, the expression of ASC, NLRP3 were detected by Western blot. **K**, **L** FLS-RA were treated with nigericin by the concentration of 10 µM for 24h accompanied with Activin (inducer of SMAD2) or not, the expression of ASC, NLRP3 were detected by Western blot. The experiment was repeated three times and Statistical data was presented as mean ± SD. In **A**–**E** and **G**, * means comparison between control and nigericin groups. # means comparison between nigericin and nigericin+SMAD2 groups. In **J**, * means comparison between control and nigericin groups. # means comparison between nigericin and nigericin+SB505124 groups. ns *P*>0.05 * ,# *P*<0.05

The supernatant was collected and the ELISA assay demonstrated that the content of inflammatory factors IL-1β, IL-18, IL-6, and IL-8 was decreased (Fig. 5A–D; *P*<0.05). Moreover, the release of LDH was significantly down-regulated in the LV-SMAD2-treated group (Fig. 5E; *P*<0.05). The above results indicated that overexpression of SMAD2 alleviated the pyroptosis of FLS-RA and the secretion of inflammatory factors.

### SMAD2 affected FLS-RA pyroptosis via the TGF-β pathway

TGF-β pathway is important in the transcription of multiple gene and normal cell function. SMAD2 is the major effector molecule in the TGF-β signaling pathway. Trans-phosphorylation by TGF-β receptor II and TGFβ receptor I activate SMAD2 and SMAD3 through phosphorylation at specific Ser residues in their C-terminal regions. Therefore, we detected the expression of TGF-β, phosphorylated-SMAD2 (p-SMAD2), and total SMAD2, TGF-β, and p-SMAD2 and total SMAD2 were up-regulated in the nigericin- and LV-SMAD2-treated group (Fig. 5F, G; *P*<0.05). The ratio of p-SMAD2/total SMAD2 was shown in Fig. 5H. Besides, we pre-treated FLS-RA with SB505124 (inhibitor of TGF-β receptor I), ASC and NLRP3 displayed increased expression compared with nigericin treated group (Fig. 5I, J; *P*<0.05). We also pre-treated FLS-RA with Activin, ASC, and NLRP3 did not decrease significantly compared with the nigericin-treated group (Fig. 5K, L; *P*<0.05). Briefly, SMAD2 declined FLS-RA pyroptosis via the TGF-β pathway.

### Increasing SMAD2 expression alleviated inflammatory and cartilage destruction in CIA rats

**Fig. 6:**
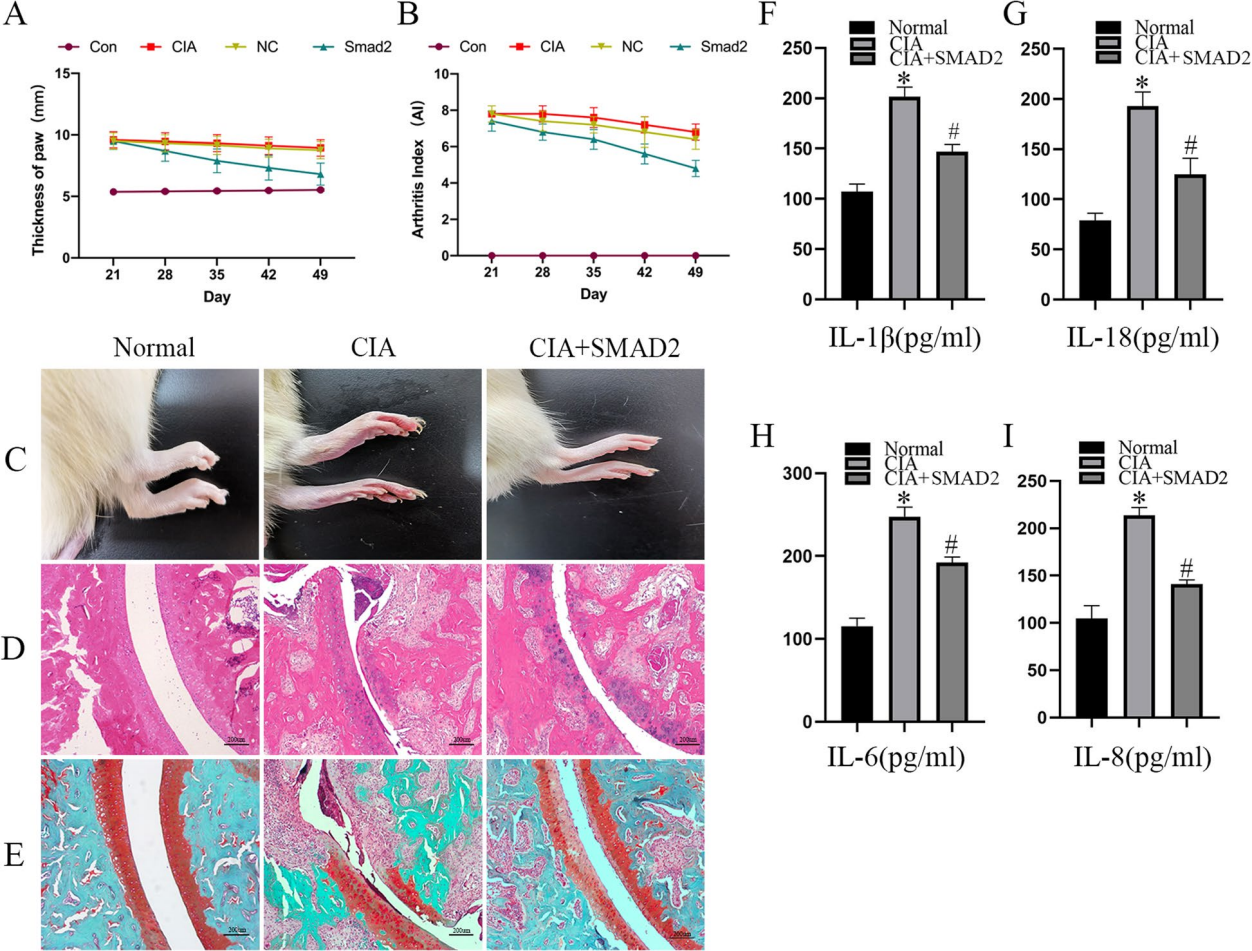
Increasing SMAD2 expression alleviated inflammatory and cartilage destruction in CIA rats. CIA rat RA model was established and treated with LV-SMAD2. **A** Thickness of the hind paw of rats in each group. **B** Arthritis index of rats in each group. **C** Representative photographs of the hind paw of rats in each group. **D** HE staining of ankle joint sections. **E** Safranin O/fast green staining of ankle joint sections. **F**–**I** Content of inflammatory cytokines IL-1β, IL-18, IL-6, and IL-8 in the serum of rats was determined by ELISA. The experiment was repeated three times and statistical data was presented as mean ± SD. In **F**–**I**, * means comparison between control and CIA groups. # means a comparison between CIA and SMAD2 groups. * ,# *P*<0.05

RA rats were conducted by injection of type II collagen. Four weeks after the first immunization, rats were intra-articularly injected with LV-negative control and LV-SMAD2. As shown in Fig. 6A, compared with the control group, CIA rats treated with LV-SMAD2 presented visibly decreased limb redness and swelling. CIA rats treated with LV-SMAD2 showed obvious improvement in arthritis index (Fig. 6B). Similar results were also observed in the representative photographs taken of the ankle joint morphology (Fig. 6C). The therapeutic effect of SMAD2 was further evaluated by detecting inflammation and cartilage and bone erosion of ankle joint sections using H&E staining and Safranin O/fast green staining. There is no inflammation or erosion of cartilage in normal rats. CIA rats displayed significant cartilage destruction and the intact articular cavity was narrow. In comparison, synovial inflammation was substantially reduced and cartilage degradation was alleviated in the LV-SMAD2-treated groups (Fig. 6D). Similar results were observed in Safranin O/fast green staining of ankle joint sections (Fig. 6E). To explore the effect of SMAD2 on inflammatory cytokines in CIA rats, the IL-1β, IL-18, IL-6, and IL-8 expressions in serum of CIA rats with or without LV-SMAD2 treatment were detected by ELISA. The results were shown in Fig. 6F–I, and inflammatory cytokine expression in rats from the LV-SMAD2 group was obviously decreased compared to the ones from the control group. In brief, increasing expression of SMAD2 alleviated inflammatory and cartilage destruction in CIA rats.

## Discussion

RA is an autoimmune disease characterized by synovitis and irreversible destruction of cartilage and bone. During the development of RA, the synovium becomes hyperplastic and invasive [20]. The main cells in the lining layer of the synovial membrane are FLS and synovial tissue macrophages [21]. RA-FLS show tumor-like characteristics, such as abnormal proliferation, migration, and invasion. Besides, its ability to secrete chemokines and cytokines is enhanced, and an unbalanced network of cytokines contributes to inflammatory response, eventually developing into RA [22]. In this study, we collected synovial tissues from patients with severe joint trauma and RA patients undergoing TKA, the protein, and RNA of SMAD2 were down-regulated in RA groups. We also cultured primary FLS, and the expression of SMAD2 was consistent with that in synovial tissues (Fig. 1). Hence, we hypothesized that SMAD2 may involve in pathological changes of FLS during the pathogenesis of RA.

Pyroptosis is a kind of programmed cell death, characterized by cell membrane rupture, release of inflammatory cytokines, and activation of inflammatory response. Pyroptosis depends on activated inflammasomes, which can promote procaspase-1 cleaved into active caspase-1, mature and release of IL-1β, IL-18, and cleavage of GSDMD [23–25]. It is reported that ultra pyroptosis of FLS is closely related to the destruction of cartilage and bone and persistent inflammation in RA, NLRP3 inflammasomes (NLRP3, ASC, and caspase-1 complex) participate in the process mentioned before [14]. Gen Li et al. reported that miR-155 inhibits chondrocyte pyroptosis by targeting SMAD2 in knee OA [18], while there was no evidence of whether SMAD2 participates in FLS pyroptosis in RA. Since we have found that the expression of SMAD2 was decreased in RA, we transfected LV-SMAD2 in FLS-RA, and induced FLS pyroptosis by nigericin. NLRP3 inflammasomes (NLRP3, ASC, and caspase-1 complex) and N terminal of GSDMD were decreased, as shown in Fig. 2. To further demonstrate the effect of SMAD2 on FLS pyroptosis, we performed flow cytometry analysis, percentage of 7− AAD+ annexin V+ late apoptotic cells and caspase-1 positive cells were also decreased. Pyroptosis morphological changes of FLS significantly alleviated by electron microscope. IL-1β and IL-18 belong to IL-1 family, which can mediate the expression of IL-6, IL-8, and TNF-α via the NK-κB pathway [26]. IL-1β and IL-18 are located in the cytoplasm at rest, which are converted into mature when caspase-1 is activated. They are transported outside of cells with the help of GSDMD [27]. In this study, SMAD2-treated FLS-RA exhibited decreased secretion of IL-1β, IL-18, IL-6, and IL-8. All data above implied that SMAD2 declined FLS pyroptosis by inhibition of NLRP3 inflammasomes.

SMAD2 is the major effector molecule in the TGF-β signaling pathway, upon ligand binding, the type II receptor phosphorylates the type I receptor, then the type I receptor induces the phosphorylation of the two C-terminal serines on SMAD2 (Ser465 and Ser467) [28]. The dephosphorylated of C-terminal serines of SMAD2 leads to the attenuation of TGF-β signaling. In our study, C-terminal serines phosphorylated SMAD2 (Fig. 5F) and TGF-β were increased in the LV-SMAD2-treated FLS-RA compared with that in the nigericin-treated group. The ratio of p-SMAD2/total SMAD2 was higher in LV-SMAD2-treated FLS-RA compared with that in the nigericin-treated group, which means p-SMAD2 appears to be increasing not simply because the overall expression of SMAD2 was increasing. Besides, SB505124 (inhibitor of TGF-β receptor I) further increased the expression of ASC and NLRP3. On the other hand, Activin (inducer of SMAD2) did not affect the expression of ASC and NLRP3 significantly. Therefore, we speculated that SMAD2 affected FLS-RA pyroptosis via the TGF-β pathway. In vivo experiments on CIA rats further confirmed the results mentioned before that overexpression of SMAD2 by local intra-articular injection of LV-SMAD2 can effectively alleviate the symptoms of CIA rats and eased the inflammatory factors in serum. While the regulation of pyroptosis is complex, there are two kinds of activation patterns during pyroptosis, including classical pathways, mediated by NLRP3 inflammasomes, and non-classical pathways, mediated by bacterial lipopolysaccharide (LPS) and caspase-4/5/11 [23–25]. Our study only focuses on SMAD2 regulating pyroptosis by classical pathways, whether SMAD2 can regulate pyroptosis by non-classical pathways has not been described. In addition, SMAD2 can also be phosphorylated by extracellular signal-regulated kinase 1 (ERK1) at Thr8 on the N-terminal and this post-translation modification leads to increased SMAD2 stability [29]. Whether SMAD2 can regulate pyroptosis and secretion of inflammatory factors via other pathways such as MAPK need to be further explored. Besides, the FLS were collected from rheumatoid arthritis patients undergoing knee arthroplasty in our study, these patients suffered from severe pain and bone destruction. Whether SMAD2 decreased and pyroptosis of FLS enhanced in the early stage of rheumatoid arthritis similar to our results is still unclear, we will investigate this further in the future.

## Conclusion

In summary, our study found that SMAD2 inhibited FLS-RA pyroptosis by down-regulating of NLRP3 inflammasomes (NLRP3, ASC, and caspase-1 complex) and eased the secretion of inflammatory factors via the TGF-β signaling pathway. This study suggests that SMAD2 may be a potential therapeutic target for RA in the future.

### Supplementary Information

**Additional file 1.**

## Data Availability

Data are available from the corresponding author on reasonable request.

## Abbreviations

RA: Rheumatoid arthritis
FLS: Fibroblast-like synoviocytes
IL: Interleukin
DMARDs: Disease-modifying anti-rheumatic drugs
NLRP3: Nucleotide-binding oligomerization segment-like receptor family 3
GSDMD: Gasdermin-D
LDH: Lactate dehydrogenase
TGF-β: The transforming growth factor β
CIA: Collagen-induced arthritis
