# Supplementary figures and images for: SMAD2 inhibits pyroptosis of fibroblast-like synoviocytes and secretion of inflammatory factors via the TGF-β pathway in rheumatoid arthritis
Source: Arthritis Res Ther. 2023 Aug 9;25:144. doi: 10.1186/s13075-023-03136-1 (PMC10410963; doi:10.1186/s13075-023-03136-1)

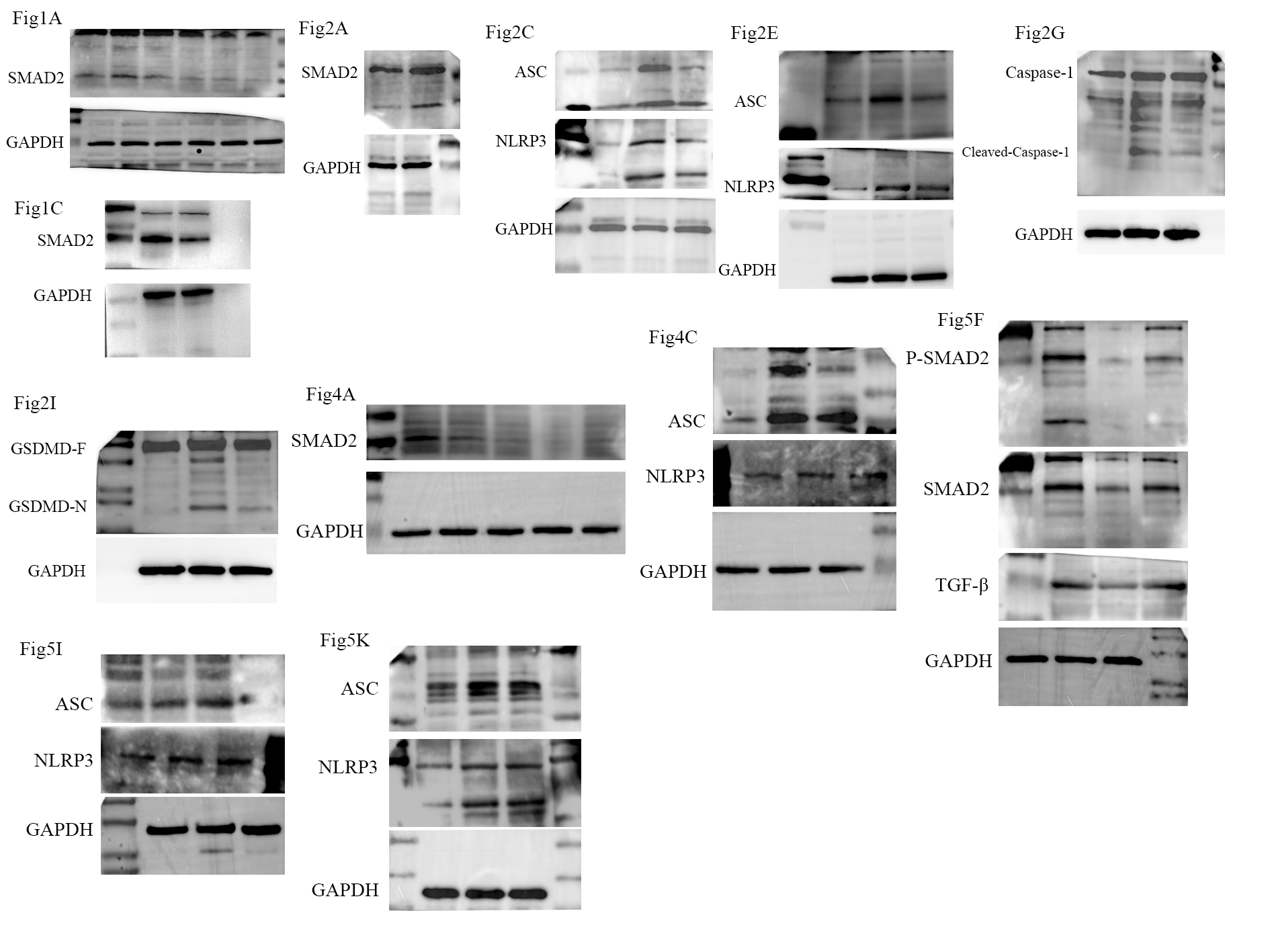

Supplement: Supplementary file 1 — Additional file 1. [file 13075_2023_3136_MOESM1_ESM.tif]
